# Dysregulation of Type II Transmembrane Serine Proteases and Ligand-Dependent Activation of MET in Urological Cancers

**DOI:** 10.3390/ijms21082663

**Published:** 2020-04-11

**Authors:** Shoichiro Mukai, Koji Yamasaki, Masato Fujii, Takahiro Nagai, Naoki Terada, Hiroaki Kataoka, Toshiyuki Kamoto

**Affiliations:** 1 Department of Urology, Faculty of Medicine, University of Miyazaki, Miyazaki 889-1692, Japan; koji_yamasaki@med.miyazaki-u.ac.jp (K.Y.); masato_fujii@med.miyazaki-u.ac.jp (M.F.); takahiro_nagai@med.miyazaki-u.ac.jp (T.N.); naoki_terada@med.miyazaki-u.ac.jp (N.T.); tkampro@med.miyazaki-u.ac.jp (T.K.); 2 Oncopathology and Regenerative Biology Section, Faculty of Medicine, University of Miyazaki, Miyazaki 889-1692, Japan; mejina@med.miyazaki-u.ac.jp

**Keywords:** matriptase, hepsin, hepatocyte growth factor (HGF), MET, prostate cancer, renal cell carcinoma, bladder cancer, HGF activator inhibitor (HAI)

## Abstract

Unlike in normal epithelium, dysregulated overactivation of various proteases have been reported in cancers. Degradation of pericancerous extracellular matrix leading to cancer cell invasion by matrix metalloproteases is well known evidence. On the other hand, several cell-surface proteases, including type II transmembrane serine proteases (TTSPs), also induce progression through activation of growth factors, protease activating receptors and other proteases. Hepatocyte growth factor (HGF) known as a multifunctional growth factor that upregulates cancer cell motility, invasiveness, proliferative, and anti-apoptotic activities through phosphorylation of MET (a specific receptor of HGF). HGF secreted as inactive zymogen (pro-HGF) from cancer associated stromal fibroblasts, and the proteolytic activation by several TTSPs including matriptase and hepsin is required. The activation is strictly regulated by HGF activator inhibitors (HAIs) in physiological condition. However, downregulation is frequently observed in cancers. Indeed, overactivation of MET by upregulation of matriptase and hepsin accompanied by the downregulation of HAIs in urological cancers (prostate cancer, renal cell carcinoma, and bladder cancer) are also reported, a phenomenon observed in cancer cells with malignant phenotype, and correlated with poor prognosis. In this review, we summarized current reports focusing on TTSPs, HAIs, and MET signaling axis in urological cancers.

## 1. Introduction

The interaction between cancer cells and stromal cells in the pericellular microenvironment has been fully discussed in the literature [1,2]. On the cancer cell surface, a variety of receptors for cytokines, growth factors, and protease activating-type receptor are widely expressed. The corresponding ligands (such as cytokines and growth factors) are provided from blood cells, stromal cells, and cancer cells in various situations. Among these, several growth factors must be proteolytically activated to achieve the biological activities. Recently, the activating process has been clarified. For example, hepatocyte growth factor (HGF) is secreted as an inactive zymogen (pro-HGF) by stromal fibroblasts and converted to active mature form by proteolytic cleavage [2,3,4]. HGF activator (HGFA) was initially identified and considered to be the single major activator in serum [3,4,5,6,7]. Indeed, coagulation cascade is initiated in pericancerous lesion, and then HGFA changes to the active form by thrombin-induced proteolytic activation [3,4,5,6,7]. Subsequently, several cell-surface transmembrane-type activators, including hepsin and matriptase, were identified [8,9,10,11,12]. These proteases may also be candidates for major activators considering HGF behavior (the so-called sticky protein) and the functional location (MET is located on the cell membrane). In physiological condition, the activation is tightly regulated by specific cell-surface protease inhibitors such as HGFA inhibitors (HAIs). However, dysregulated overactivation of pro-HGF has been reported to aggressively induce cancer invasion and metastasis through increased phosphorylation of MET [10,11,12]. This review focuses on the cell-surface pro-HGF activation system in urological cancers, including prostate cancer (PC), renal cell carcinoma (RCC), and urothelial carcinoma (UC) of bladder, and summarizes its significance in the progression of cancer.

## 2. HGF/MET and the Related Molecules

### 2.1. HGF and MET in Cancer

#### 2.1.1. HGF/MET Signaling Axis

**Figure 1:**
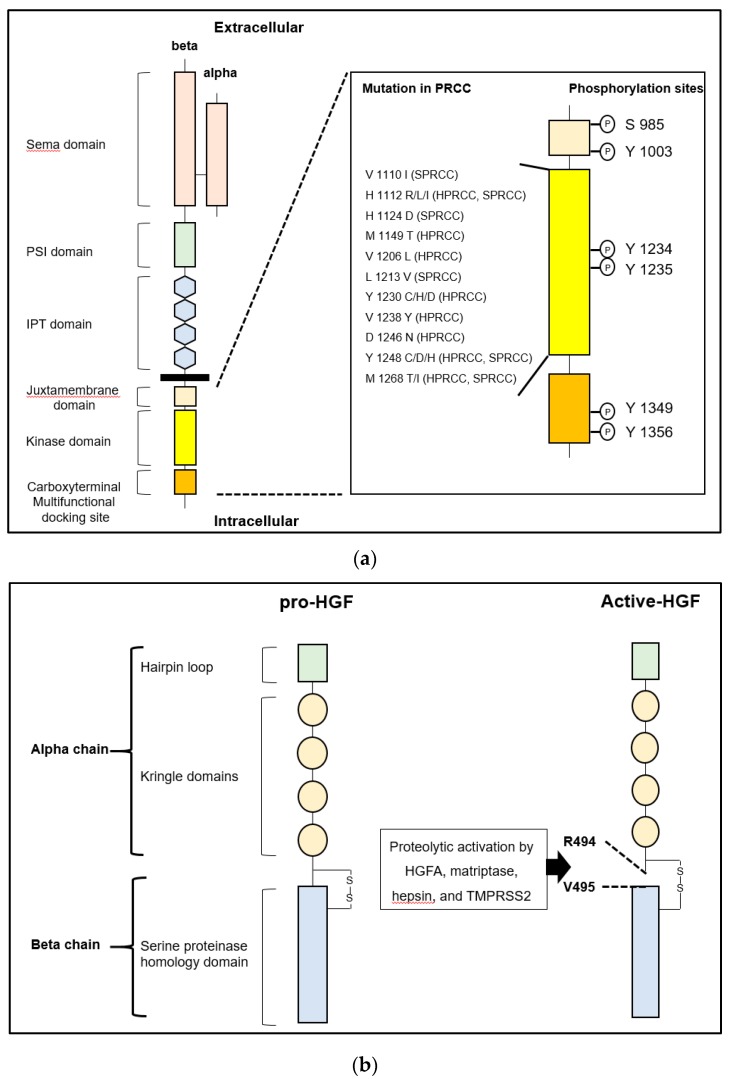
(**a**) Left: The structure of human MET is shown. MET consists of extracellular alpha and single-pass transmembrane beta chain, which are disulfide-linked heterodimer. The beta chain is composed of six major domains including Sema (semaphorin), PSI (plexin, semaphorin, integrin), IPT (immunoglobulin-like regions in plexins and transcription factors), juxtamembrane, tyrosine kinase domain, and multifunctional docking site. Right: Sites of point mutation in hereditary and sporadic papillary renal cell carcinoma (HPRCC and SPRCC) and conventional phosphorylation sites in intracellular domains are shown. (**b**) Left: The structure of human pro-hepatocyte growth factor (HGF) is shown. HGF consists of four Kringle domains and a serine proteinase homology domain. Right: The active form of HGF is shown. HGFA, hepsin, matriptase, and TMPRSS2 proteolytically cleave between Arg 494 and Val 495 to convert to a two-chain heterodimeric active form. One-letter abbreviation of amino acids is used.

MET, encoded by *Met* proto-oncogene located on chromosome 7q31, is a tyrosine kinase-type specific receptor of HGF, which forms disulfide-inked heterodimer consisting of an extracellular alpha chain and single-pass transmembrane beta chain [3,13,14,15]. As shown in Figure 1, the intracellular domain of the beta chain comprises a juxtamembrane domain and catalytic kinase domain containing an activation loop and carboxy-terminal multifunctional docking site. The juxtamembrane domain downregulates the kinase activity by phosphorylation of Ser975, while the catalytic kinase domain upregulates the activity by phosphorylation of Tyr1234 and Tyr1235. The multifunctional docking sites contain Tyr1349 and Tyr1356, which lead to downstream signaling through several intracellular adaptor proteins [3,13,14,15,16]. Increased expression of MET with worse prognosis has been reported in various cancer cells, and phosphorylation (activation) potently promotes invasion and metastasis [16,17,18,19]. Activation of HGF/MET signaling axis in cancer cells also plays a significant role in proliferation, angiogenesis, epithelial-mesenchymal transition (EMT), and drug resistance [3,13,14]. Activation is introduced by: 1) ligand (HGF)-dependent activation, 2) reciprocal activation by overexpression-induced MET oligomerization, 3) activating point mutation of tyrosine kinase domain, and 4) transactivation by heterodimerization with another receptor tyrosine kinase [3,13,14]. In the ligand-dependent activation, proteolytic activation of pro-HGF is necessary. As mentioned above, two major activating protease families were reported: 1) a serum serine protease, HGFA; and 2) type II transmembrane serine proteases (TTSPs) such as matriptase, hepsin, and transmembrane protease/serine (TMPRSS) 2 [3,10,11,12]. Although these pro-HGF activating proteases are tightly regulated by two transmembrane serine protease inhibitors, HAI-1 and HAI-2, downregulation of HAIs has been observed in several cancers and has been shown to induce progression [11,12].

### 2.2. Cell Surface pro-HGF Activating Enzymes and the Regulators

#### 2.2.1. Type-II Transmembrane Serine Proteases (TTSP) in Cancers

**Figure 2:**
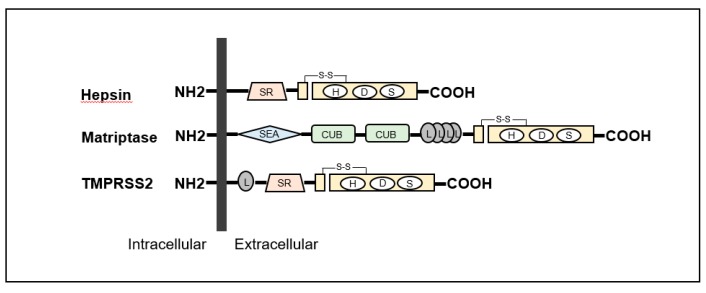
Structures of hepsin, matriptase and transmembrane protease serine (TMPRSS) 2 are shown. All type II transmembrane serine proteases (TTSPs) show single-pass transmembrane proteins with intracellular NH2-terminus and extracellular carboxy-terminal serine protease domains. Hepsin is composed of scavenger receptor (SR) and serine protease domains. Matriptase contains sea urchin sperm protein/enteropeptidase/agrin (SEA) domain, Cls/Cls, urchin embryonic growth factor, bone morphologic protein-1 (CUB) domain, four low-density lipoprotein receptor (L) domains class A and serine protease domains. TMPRSS2 is consists of an L domain, SR, and serine protease domains.

The TTSP family in humans consists of 17 serine proteases [3,10,17]. The structures are specified as a single-pass hydrophobic transmembrane domain near the N-terminus with a short intracellular domain and a large extracellular portion including a carboxy-terminal serine protease domain [3,10,17]. All TTSPs are divided into the four subfamilies of hepsin, matriptase, human airway trypsin-like protease (HAT) and corin (Table 1) [3,10,17]. All TTSPs belong to the S1 peptidase family (noted in MEROPS as clan PA, family S1), and a catalytic triad consists of serine, aspartate, and histidine residues, as shown in Figure 2 [20,21]. Hepsin, matriptase and TMPRSS2 shows a strong cleavage preference for substrate with arginine in the P1 position [20,21]. In urogenital cancers, the expression of matriptase, hepsin and TMPRSS2 has been reported (Figure 2). Therefore, we focused on these TTSPs in this review.

#### 2.2.2. Matriptase

*Matriptase (MT-ST1, ST14)* gene is located on human chromosome 11q24-25, and 855 amino acids are encoded in the gene [3,10,17,18]. The molecular weight of matriptase is 80–90-kda. Matriptase was first discovered in breast cancer cell line (T-47D) and purified from human milk [3,10,17,18]. It is expressed in human epithelial cells of various organs to maintain the formation of epithelial barrier formation [3,10,11,12,19]. In addition, the major enzymatic functions are reported as follows: 1) activation of hepatocyte growth factor zymogen (pro-HGF), pro-platelet-derived growth factor (PDGF)-C, -D, and pro-macrophage stimulating protein (MSP); 2) activation of protease-activated receptor (PAR)-2; 3) activation of urokinase-type plasminogen activator; 4) degradation of extracellular matrix; and 5) activation of prostasin, which is a glycosylphosphatidylinositol (GPI)-anchored protease known to activate epithelial sodium channel (ENaC) [3,10,17,18]. Among TTSPs, matriptase has been reported as the most efficient activator of pro-HGF [3,10,11,12]. HAIs are major regulators of matriptase, and deregulation of matriptase activity facilitates cancer progression [3,10,11,12]. Indeed, matriptase expression is reported to be upregulated in various cancers (breast, ovarian, uterine, colon, cervical, skin, pancreatic, esophageal, head and neck, prostate, bladder and renal cell carcinoma: RCC) with poor prognosis [3,10,11,12,22].

#### 2.2.3. Hepsin

*Hepsin* (*HPN, TMPRSS1*) gene is located on human chromosome 19q13.11, and 417 amino acids are encoded [3,10,17,18]. The molecular weight of hepsin protein is 45-kda. Although mRNA is highly expressed in liver and kidney, ubiquitous expression of the protein is reported [3,10,23]. The functions are reported as follows: 1) activation of pro-HGF; 2) activation of pro-MSP; 3) activation of pro-urokinase-type plasminogen activator; and 4) cleavage of laminin-332 [24]. Similar to matriptase, the catalytic activities of hepsin are regulated by HAI-1 and HAI-2 [3,10,11,12]. In cancer, overexpression of hepsin mRNA is reported in prostate, ovary, kidney, and breast [3,10,17,18]. Increased expression of the protein is also reported in prostate, ovarian, breast, and endometrial cancer [3,10,17,18].

#### 2.2.4. Regulators of TTSPs—HAIs

**Figure 3:**
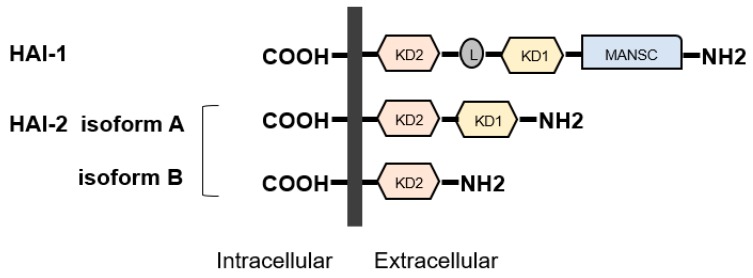
Structures of hepatocyte growth factor activator inhibitor (HAI)-1 and HAI-2 are shown. HAIs show single-pass transmembrane protein with intracellular carboxy-terminus and extracellular specific protease inhibitor domains, the so-called Kunitz domain (KD). HAI-1 is composed of two KDs, L domain, and motif at N terminus with seven cysteines (MANSC) domains. There are two isoforms in HAI-2. Similar to HAI1, HAI-2 isoform A has two KDs, whereas isoform B has a single KD.

*HAI-1* (*SPINT-1*) gene is located at 15q 15.1 and *HAI-2* (*SPINT-2*) is located at 19q 13.2 [3,11,12]. Both proteins were initially identified in conditioned medium of human gastric cancer cell line MKN45 [3,11,12,25,26]. HAI-2 was also purified as placental bikunin from placenta [3,11,12,27]. The proteins have two specific extracellular Kunitz-type serine protease inhibitor domains, (KD)-1 and KD-2, except for a splicing variant of HAI-2 (isoform B has single KD) (Figure 3), which can inhibit several trypsin-like serine proteases, including all pro-HGF-activating enzymes [3,11,12,28,29]. Whereas, HAIs were initially discovered as HGFA inhibitors, they also inhibit matriptase and hepsin [3,11,12]. In addition, HAIs are required for intracellular transport and cell surface localization of matriptase in several types of cells [3,10,11,12]. HAI-1 is reported to express in the majority of normal epithelial cells [3,11,12,30]. In physiological condition, HAI-1 maintains epithelial integrity through regulation of matriptase activity [3,11,12,30]. HAI-1 is also required for placental differentiation, embryonic development and postnatal survival [11,12,31]. However, it has been reported that insufficient expression revealed dysregulation of pro-HGF activating enzymes in various cancers leading to progression [11,12]. Indeed, decreased expression of HAI-1 induced carcinogenesis (skin, intestine) and progression with worse prognosis (gastrointestinal, breast, ovarian, endometrial cancers and RCC) [11,12,32,33,34,35,36,37,38,39,40,41,42]. In addition, HAI-1 is also known as a suppressor of epithelial mesenchymal transition (EMT) [43].

HAI-2 is ubiquitously expressed in normal cells, including epithelial, mesenchymal, blood cells and trophoblasts [12]. HAI-2 is reported to maintain the integrity of intestinal epithelium through regulation of matriptase-induced epithelial cell adhesion molecule (EpCAM) cleavage [44]. Downregulation by hypermethylation of *SPINT2* gene has been reported in several cancers, including hepatocellular carcinoma, RCC, melanoma, gastric carcinoma, and esophageal squamous cell carcinoma [45,46,47,48]. Expression of HAI-2 is also decreased in PC. However, no apparent *SPINT2* promoter methylation has been observed in either clinical samples or cell lines [49]. In this report, the authors suggest that posttranslational regulation of HAI-2 expression is essential in prostate cancer. The regulatory role of HAI-2 in the activation of pro-HGF by inhibiting the activating proteases (including matriptase), which induces HGF/MET signaling axis, has been considered a major suppressive function in cancer progression [11,12]. Additionally, an alternative function such as the activation of caspase 3 in esophageal squamous cell carcinoma leading to the promotion of apoptosis and inhibition of proliferation was also reported [47,50]. However, HAI-2 has also been reported to be required for invasive growth in oral squamous cell carcinoma, which suggests that the role of HAI-2 may be tissue or cell-type specific and dependent on targeting TTSPs [51].

## 3. TTSPs and HAIs in Urological Cancers

### 3.1. Prostate Cancer

#### 3.1.1. HGF/MET Signaling and Hepsin

Increased expression of MET and phosphorylation in proliferative inflammatory atrophy (PIA) and prostatic intraepithelial neoplasia (PIN), which are known precancerous findings, have been reported [52,53]. Regarding carcinogenesis, two interesting phenomena were reported in mouse models: 1) conditional *MET* overexpression in prostatic luminal cells revealed development of PIN by administration of HGF, however, the conditional transgenic mouse did not produce PC [54] and 2) Conditional overexpression of hepsin in prostate epithelium revealed disruption of basement membrane in vivo [55]. These results suggested that the single expression of an HGF-related molecule may be insufficient for carcinogenesis because co-existence of MET with PTEN loss or expression of hepsin with Myc in normal prostate epithelium revealed development of cancer [54,55,56]. However, expression of MET in PC induced aggressive metastatic phenotype with sarcomatoid features [54], and expression of hepsin in localized cancer promoted metastatic cancer with differentiation to neuroendocrine phenotype [55]. Therefore, HGF/MET signaling and hepsin may have a significant role in the progression of PC [57,58,59]. Indeed, upregulation of MET was observed in metastatic lesion compared with primary site [60]. In addition, overexpression of MET is reported in patients with metastatic PC and positively correlated with progression [3,53,61]. Furthermore, inhibition of hepsin by small molecule inhibitor has been reported to reduce bone metastasis of PC [58].

In the normal prostate, expression of MET is observed in basal cells, but not in luminal cells [52]. Androgen receptor (AR) is, however, expressed in luminal cells [52]. Of interest, the inverse expression of MET and AR has also been reported in PC [62,63]. For example, MET is highly expressed in AR-negative prostate cancer cell lines such as PC3 and DU145, however, it is downregulated in AR-positive cell lines (LNCaP, LAPC-4, CWR22, and LuCaP) [62,63]. In addition, the degree of MET expression in CWR22 cell line is reported to continue increasing during the process to become androgen independent phenotype (CWR22R), and downregulation of AR induced upregulation of MET with the signaling system is observed [62,63].

Despite this, AR signaling remains in the majority of CRPC. This is explained by the clinical efficacy of AR signaling-targeted agents (abiraterone and enzalutamide) for CRPC, and a recent clinical phase III trial of cabozantinib (dual inhibitor of MET and vascular endothelial growth factor receptor for CRPC revealed insufficient results [64]. The reported efficacy of combined therapy targeting both AR and MET suggests the coexistence of AR-positive cells and MET-positive cells in CRPC [65].

#### 3.1.2. Matriptase and HAIs

Inhibition of matriptase by synthetic small molecule inhibitor and analysis of downregulation using hammerhead ribozyme revealed reduced invasiveness and motility of PC cell lines [66]. The significant role of matriptase in cancers has been discussed along with the ratio of HAIs activity. Several studies revealed that overexpression and functional upregulation of matriptase rather than HAIs leads to cancer progression [67,68]. In PC, increased expression of matriptase with concomitant loss of HAI-1 was observed [67]. In addition, a correlation between downregulation of HAI-2 with upregulation of matriptase and increasing PC tumor grade has been reported [49,69]. However, the following evidence suggests that HAI-2 is a main regulator of matriptase [49,50,68,69,70]. The role of HAI-2 in invasiveness and metastatic potential of PC cells was analyzed using human prostate cancer progression model cell lines and the xenograft model [49,68,69]. As a result, the expression of HAI-2 but not HAI-1 in cancer cells was gradually downregulated depending on increasing invasiveness accompanying matriptase upregulation. In additional analyses, the authors confirmed that matriptase induced upregulation of PC cell motility as well as the fact that invasive and metastatic capabilities were negatively regulated by HAI-2. This phenomenon was also confirmed by the inhibition of matriptase using recombinant protein consisting of HAI-2-Kunitz domains [68,69].

#### 3.1.3. TMPRSS2 in PC

As noted above, TMPRSS2 is a member of the TTSP family. In the pathogenesis of viral infection, TMPRSS2 is known to proteolytically activate spike glycoprotein of emerging coronaviruses, including severe acute respiratory syndrome (SARS) coronavirus (CoV), SARS-CoV-2 (COVID-19), human CoV-229E, and Middle East Respiratory Syndrome CoV (CoV-EMC), which is required for virus-cell membrane fusions [71,72,73]. The proteolytic cleavage and activation of hemagglutinin protein are also essential for the viral infectivity of influenza A virus (strains of H1N1, H3N2, and H7N9) [73,74]. Recent studies revealed that a gene fusion of TMPRSS2 to erythroblast transformation-specific (ETS) transcription factors is commonly observed in prostate cancer tissues [75,76]. Among these, fusion of *TMPRSS2* and *ERG* is the most common (approximately 50%) chromosomal rearrangement [75,76]. However, no apparent proteolytic activity in this fusion protein has been reported (the protein mainly acts as a transcription factor). Expression of TMPRSS2 is positively regulated by androgens and expressed in both primary and metastatic sites of hormone sensitive PC [77,78]. Expression is reported to be increased in higher Gleason pattern [77,78]. As substrates, components of extracellular matrix (nidogen-1, laminin β1), matriptase zymogen, and pro-HGF were reported [78]. Ko et al. reported that androgen-induced TMPRSS2 can initiate a pericellular proteolytic cascade to activate matriptase, which promotes cancer cell invasion [79]. In addition, the correlation between increased expression of TMPRSS2 and increased matriptase activation in PC tissue was also confirmed. They concluded that TMPRSS2 can promote PC progression through increased matriptase activation and degradation of nidogen-1 and laminin β1 [79].

**Figure 4:**
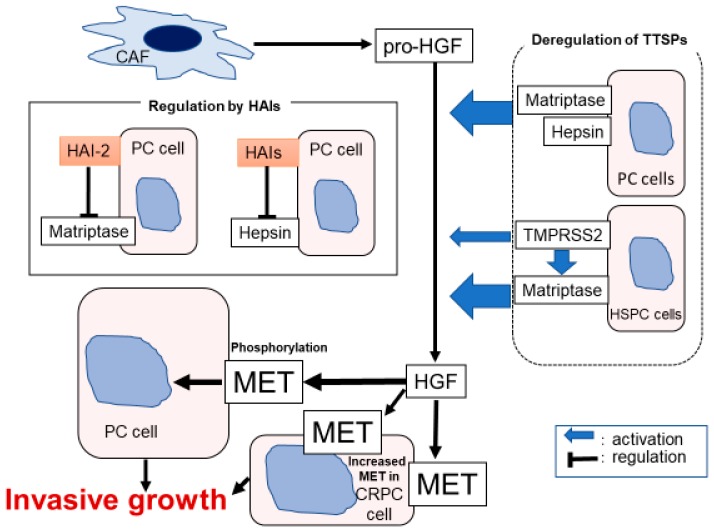
Hypothetical model of pericellular activation of hepatocyte growth factor zymogen (pro-HGF) in prostate cancer (PC) is shown. Downregulation of HAIs induced dysregulated overactivation of matriptase, hepsin, and TMPRSS2, leading to HGF-dependent phosphorylation of MET.

The evidences of Section 3.1.1, Section 3.1.2 and Section 3.1.3 are summarized in Figure 4.

### 3.2. Renal Cell Carcinoma

#### 3.2.1. HGF/MET Signaling

The most common histological subtype is clear cell RCC (approximately 80%–90% of RCC), followed by papillary RCC (10%–15%) [80]. Since expression of MET is observed in all subtypes, activating germline point mutations of MET are well known as the major cause of hereditary papillary RCC (PRCC) type-1 [15,16]. The mutation occurs at the tyrosine kinase (TK) domain and induces continuous ligand-independent phosphorylation of MET [15,16]. Although the mutation is observed only in 10%–46% of sporadic PRCC, overexpression including amplification of MET is common [81]. Of interest, phosphorylation of MET with auto-activating mutation was enhanced to a significant degree by HGF stimulation [82].

Inactivation of von Hippel-Lindau (*VHL*) tumor suppressor gene is the most common genetic abnormality of clear cell RCC [83,84]. Inactivation occurs not only in VHL patients (germline mutation) but also in patients with sporadic clear cell RCC (somatic abnormality) by deletion, mutation and epigenetic hypermethylation [83,84]. Deficiency of VHL protein in clear cell RCC causes progression of angiogenesis through activation of hypoxia inducible factor (HIF) 1α and 2α, which are major transcription factors of vascular endothelial growth factor (VEGF) and platelet-derived growth factor (PDGF) [83]. Of interest, HIF-1α is also reported to activate transcription of MET [3,84]. Indeed, overexpression of MET with worsening prognosis has been reported in patients with cc RCC. In addition, a significant body of evidence has revealed that activation of MET in RCC cells promotes proliferation, invasive activity, motility, antiapoptotic activity, and angiogenesis [3,42,84,85,86,87,88]. In addition, CD105+/CD24− cancer stem cells of RCC also expressed MET, and inhibition reduced development of bone metastasis [89].

Phase III METEOR(NCT01865747) trial revealed that cavozantinib, a novel TK inhibitor of VEGF receptor and MET, has demonstrated favorable efficacy for progression-free survival (PFS), overall survival (OS), and objective response rate (ORR) in patients with metastatic RCC in a second- or third-line treatment setting [90]. In addition, subgroup analysis of the METEOR trial demonstrated that treatment has clinical benefits in PFS, OS, and ORR for RCC patients with bone metastases associated with poor prognosis [91].

#### 3.2.2. HAIs and TTSPs in RCC

Increased expression of matriptase and hepsin was reported to be correlated with poor prognosis [3,42,92]. In addition, decreased expression of HAI-1 and HAI-2 also revealed worse prognosis in patients with RCC [3,42,45,93]. As mentioned above, hypermethylation of *SPINT2* gene in RCC has been reported [45]. Morris et al. reported that *SPINT2* promoter methylation was observed in approximately 30% of ccRCC and 40% of pRCC patients with sporadic type, and they concluded that epigenetic inactivation caused loss of tumor suppressor activities in RCC [45].

#### 3.2.3. MET and Matriptase/HAI-2 in Bone Metastasis

Approximately 30% of advanced RCC patients have bone metastasis [91,94]. Osteolytic metastasis affects patient quality of life through skeletal-related events. As mentioned above, bone metastasis is reported to be a negative prognostic factor and shows resistance to multimodal treatment, including surgery, radiation, and recent VEGFR-targeted medicines [91,94]. Interestingly, immunohistochemical analysis has revealed a significant increase in the expression of MET and matriptase in bone metastasis compared with primary site [87]. In analysis of mouse model of bone metastasis using human RCC cell line (786-O), expression of HGF, matriptase and decreased expression of HAI-2 were observed in bone metastasis compared with control [88]. Furthermore, increased phosphorylation of MET was also observed in bone metastasis [88]. These reports suggested that the HGF-dependent MET signaling axis plays a significant role in RCC bone metastasis.

**Figure 5:**
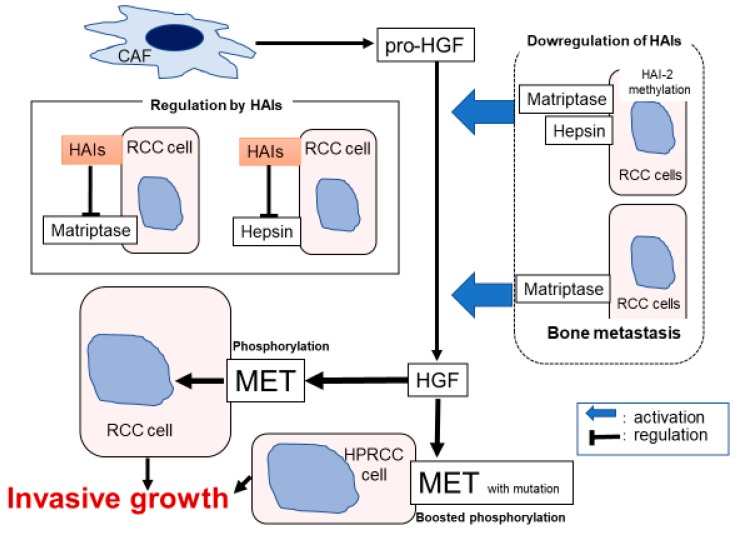
Hypothetical model of pericellular activation of pro-HGF in renal cell carcinoma (RCC) is shown. Downregulation of HAIs including HAI-2 methylation induced overactivation of matriptase and hepsin. In bone metastasis, increased expression of matriptase and downregulation of HAI-2 leads to the upregulation of HGF activation. In addition, phosphorylation of MET with auto-activating mutation was enhanced by HGF stimulation.

The evidences of Section 3.2.1, Section 3.2.2 and Section 3.2.3 are summarized in Figure 5.

### 3.3. Bladder Cancer

#### 3.3.1. Significance of HGF-dependent MET Activation in Muscle Invasive Bladder Cancer

**Figure 6:**
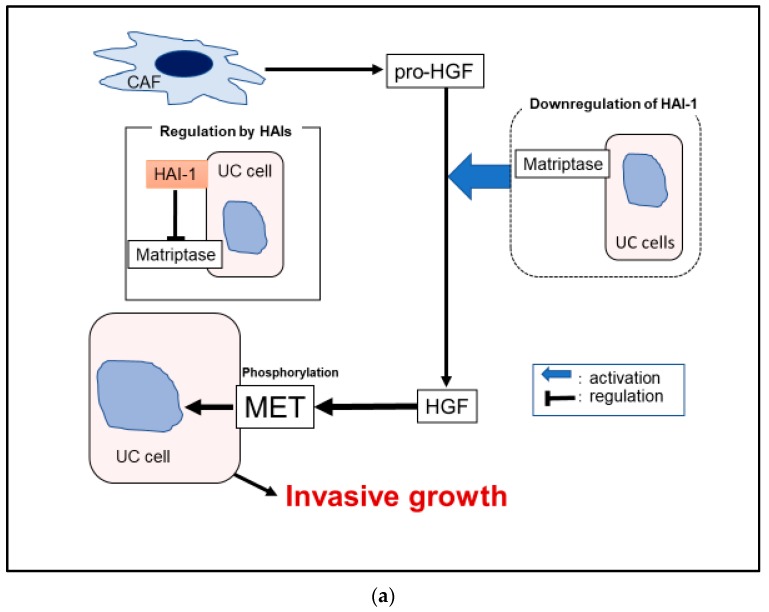

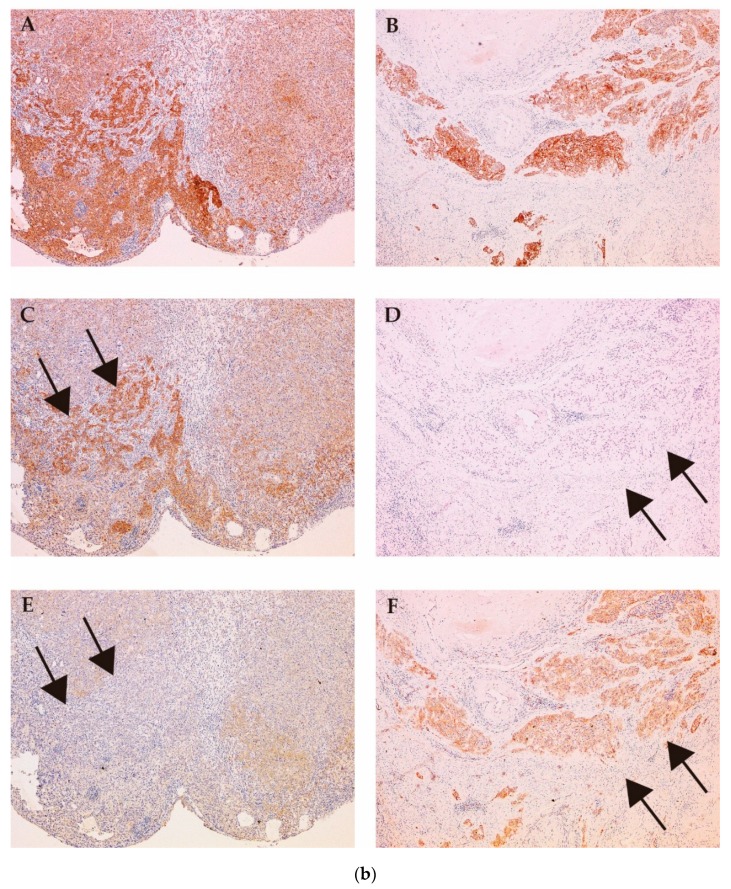
Hypothetical model of pericellular activation of pro-HGF in bladder urothelial carcinoma (UC) (**a**) is shown. Downregulation of HAI-1 induced overactivation of matriptase, which leads to upregulation of HGF-dependent MET phosphorylation (**a**). Immunohistochemical appearances are shown (**b**). Expression of MET (A–B), phosphorylated MET (C–D) and HAI-1 (E–F) is analyzed (case 1: A, C, and E; case 2: B, D, and F). Expression of MET is observed in both case 1 and case 2 (A and B). Whereas, increased phosphorylation of MET is observed in areas where expression of HAI-1 is decreased (C and E, arrows). On the other hand, phosphorylation is downregulated in areas where HAI-1 expressed (D and F, allows). The results suggested regulation of MET phosphorylation by HAI-1 may have a significant role in UC.

Pathological analysis shows that approximately 90% of bladder cancers are UC and that 5% are squamous cell carcinoma [95]. Several studies reported a positive correlation between increased expression of MET and high tumor grade, pathological staging, and survival [96,97,98,99,100]. Phosphorylation was also analyzed by immunohistochemistry [97,98]. Increased phosphorylation of Tyr1234/1235 (at activation loop) and Tyr1349 (at multi-docking site) was positively correlated with pT stage, and multivariate analysis prompted the authors to conclude that phosphorylated Tyr1349 was a significant predictive factor of pT stage only. In addition, phosphorylated Tyr1349 was correlated with expression of matrix metalloprotease (MMP)-2, MMP-7, and E-cadherin [97]. Furthermore, the relationship between increased phosphorylation of Tyr 1235 in MET and worse cancer-specific survival in patients with muscle invasive bladder cancer (MIBC) was reported by Yamasaki et al. In this study, increased expression of matriptase and decreased expression of HAI-1 were also correlated with poor prognosis [98]. Interestingly, the area of HAI expression and MET phosphorylation presented reciprocally in spite of the strong expression of both total MET and matriptase (Figure 6b) [98]. These findings suggested that HAI-1 may regulate the ligand-dependent activation of MET in patients with MIBC. As a biomarker, the utility of urinary soluble MET concentration to detect bladder cancer and MIBC was reported [101,102]. In vitro analysis, efficient suppression of invasion and proliferation by cabozantinib (dual inhibitor of MET and VEGFR) was observed in invasive UC cell lines (5637, T24) [103]. HGF/MET signaling-induced expression of MMP-1 was also suppressed by cabozantinib in the same cell lines. HGF/MET signaling-induced EMT is a major phenomenon in various cancer cells [103]. Recently, it has been reported that HGF/MET signaling-induced inhibition of SMURF2 yielded stabilization of TGF-β receptor, and that upregulation of TGF-β signaling axis leads to EMT of UC cell line [104]. However, compared with PC and RCC, the function of TTSPs and HAIs in UC is not well known. Therefore, further analysis is required.

The evidence of Section 3.3.1 is summerized in Figure 6a.

## 4. Conclusions

In this review, we summarized the significance of pericellular pro-HGF activation in PC, RCC, and bladder cancer. Overactivation (induced by increased HGF-activating TTSPs or decreased HAIs) revealed cancer progression through MET phosphorylation.

Amplification of *MET* in lung cancer is a well-known mechanism of resistance to epidermal growth factor receptor-targeted tyrosine kinase inhibitors (EGFR-TKIs) [105,106,107]. In addition, overexpression of HGF is observed in approximately 61% of patients with EGFR-TKIs-resistant lung cancer and is known as a cause of acquired resistance [106,107]. The MET-signaling pathway is a major target in these patients. However, resistance to anti-MET therapy is also promoted by high amounts of pericellular HGF [106,107]. Therefore, the pro-HGF- activation system is significant in cancer progression. In urological cancers, several small molecule inhibitors targeting MET have been used in PC and RCC in clinical trials, and cavozantinib is used as a major agent targeting MET and VEGFR in patients with advanced RCC [90,91,108,109]. In addition, the efficacy of several agents for HGF and the activating TTSPs, including anti-HGF antibody, antibodies and small molecule inhibitors of hepsin and matriptase, has been reported [3,40,110,111,112,113]. Unfortunately, however, no such specific inhibitors have been verified in clinical trials [3,10]. Both matriptase and hepsin have other independent functions besides HGF-activation [114]. Therefore, inhibition of these TTSPs may yield additional therapeutic benefits in cancer treatment. As a candidate, the inhibitor domain of HAIs (KD1) shows significant inhibition for matriptase, hepsin, and HGFA [3,11,12,69]. Therefore, we believe KD1 has favorable therapeutic potential in urological cancers.

## Figures and Tables

**Table 1:** Type II transmembrane serine protease (TTSP) family content.

Subfamily	Protease
HAT/DESC	HAT
	DESC1
	TMPRSS 11A
	HAT-like 4
	HAT-like 5
Hepsin/TMPRSS	Hepsin (TMPRSS1)
	TMPRSS 2
	TMPRSS 3
	TMPRSS 4
	TMPRSS 13
	Enteropeptidase
	Spinesin
Matriptase	Matriptase
	Matriptase 2
	Matriptase 3
	Polyserase
Corin	Corin

TTSP: type II transmembrane serine protease; HAT: human airway trypsin-like protease; DESC: differentially expressed in squamous cell carcinoma; TMPRSS: transmembrane protease, serine.

## Abbreviations

HGF	hepatocyte growth factor
HGFA	hepatocyte growth factor activator
HAI	hepatocyte growth factor activator inhibitor
PC	prostate cancer
RCC	renal cell carcinoma
EMT	epithelial-mesenchymal transition
TTSP	type II transmembrane serine protease
TMPRSS	transmembrane protease, serine
PRCC	papillary renal cell carcinoma
PDGF	platelet-derived growth factor
PAR	protease activated receptor
ENaC	epithelial sodium channel
RNA	ribonucleic acid
KD	Kunitz-type serine protease inhibitor domain
EPCAM	epithelial cell adhesion molecules
PIN	prostatic intraepithelial neoplasia
AR	androgen receptor
CRPC	castration resistant prostate cancer
TK	tyrosine kinase
VHL	von Hippel-Lindau
MMP	matrix metalloprotease
UC	urothelial carcinoma
SMURF	SMAD specific E3 ubiquitin protein ligase 2
TGF	transforming growth factor
